# lncRNA GAU1 Induces GALNT8 Overexpression and Potentiates Colorectal Cancer Progression

**DOI:** 10.1155/2021/5960821

**Published:** 2021-06-17

**Authors:** Xuemei Tang, Haoyu Ruan, Liu Dong, Sihan Li, Zhiyuan Wu, Ming Guan

**Affiliations:** ^1^Department of Laboratory Medicine, Huashan Hospital, Shanghai Medical College, Fudan University, Shanghai 200040, China; ^2^Center for Pharmacogenetics, Department of Pharmaceutical Sciences, University of Pittsburgh, PA 15261, USA

## Abstract

lncRNA is a key epigenetic regulator in biological processes. In the human cancer transcriptome library MiTranscriptome, we identified *GAU1* as the top upregulated lncRNA in colorectal cancer (CRC) by sample set enrichment analysis (overexpression ranking percentile = 99.75%, *P* < 10^−50^), which is coexpressed with the potential oncogene *GALNT8* (Spearman rho = 0.67, *P* = 2.44 × 10^−23^, TCGA dataset *n* = 184). Experimental data revealed that *GAU1* regulates the expression of *GALNT8*. The overexpression of either *GAU1* or *GALNT8* significantly promotes the cell cycle and proliferation of CRC cell lines and correlates with poor prognosis in patients with CRC (*P* = 3.04 × 10^−2^), while silencing of *GAU1* or *GALNT8* suppressed the cancer cell proliferation and induced the CRC cell line resistance to oxaliplatin *in vitro* treatment. Our results suggested that the previously less studied *GAU1* and *GALNT8* may play as CRC prognosis markers and potential targets for chemotherapy treatment.

## 1. Introduction

Colorectal cancer (CRC) ranked the third common type of cancer, adding up 10% of all cases [1]. In 2018, there were over one million new cases and over half million deaths from the disease [2]. Genetic mutations in *APC* [3], *TP53* [4], and *K-RAS* [5] have been intensively studied as major contributors to the tumorigenesis of CRC. Besides, nongenetic risk factors like aging and lifestyle also induce the development of CRC cases. However, this nonmutational alteration in CRC was less studied [6]. Massive parallel sequencing facilitated the genome-wide characterization of the human cancer transcriptome and identified long noncoding RNA (lncRNA) expression as the most common transcriptional alteration in cancer [7]. Our previous reports revealed that lncRNAs are extensively involved in the CRC development [8] and drug resistance [9], indicating that more efforts should be encouraged to identify the CRC-specific lncRNA expression and to link the biological “operator” regulating these noncoding “regulators.”

RNA-Seq technology empowered by sequence alignment and assembly provides a revolutionary approach for the prediction of full-length transcripts from both the intergenic “gene desert” and protein-coding loci [10, 11]. The MiTranscriptome database applied ab initio assembly to 7,256 curated RNA-Seq libraries from tumor, normal tissue, and cell lines so as to provide an unbiased method for gene discovery [12]. Here, by incorporating this ab initio assembly-based human cancer transcriptome database and experimental validation, we identified a colorectal cancer-related lncRNA *GAU1* from 12,382 cancer-associated lncRNA transcripts and verified its procancer function as upregulating the mRNA expression of polypeptide N-acetylgalactosaminyl transferase GALNT8, whose overexpression correlates with the cancer cell proliferation and poor patient survival.

## 2. Materials and Methods

### 2.1. Identification of *GAU1* as the CRC-Related lncRNA

The normalized counts of 12,382 ab initio-assembled lncRNA transcripts and library information of 6,476 RNA-Seq libraries (5,724 cancer-related samples and 752 normal samples) including 5,602 TCGA cases were downloaded from the MiTranscriptome website (http://MiTranscriptome.org/download/MiTranscriptome.expr.counts.tsv.gz).

Sample set enrichment analysis (SSEA) [12] was performed to test if a transcript is differentially expressed between the cancer and noncancer samples in an empirical ranking method. In brief, a weighted KS test was performed as gene set enrichment analysis (GSEA) [13] to generate the enrichment score (ES) describing the enrichment of the sample set among all tested samples. SSEA was further performed 1,000 times with random permutation of the ample labels for a set of null ES and the nominal *P* value of relative rank of observed ES within the null ES. The hypothesis testing was performed by comparing the tested ES to the null normalized enrichment score (NES) for all transcripts in a sample set. SSEA percentile score was generated by ranking the transcripts in each analysis by their NES. The tissue-type information of each transcript was obtained from the MiTranscriptome browser (http://MiTranscriptome.org).

To perform *GAU1* coexpression analysis, the normalized RSEM-FPKM mRNA expression of 382 TCGA CRC samples was obtained from TCGA firehose legacy (https://gdac.broadinstitute.org/). After sample overlapping with the MiTranscriptome database, Spearman's rank correlation coefficient of *GAU1* and all 19,815 protein-coding gene mRNA expression was calculated in 184 TCGA CRC samples.

### 2.2. Clinical Samples and Tissue Microarray

Primary CRC tissues and paired adjacent tissues were collected from 66 CRC patients. All these samples were obtained between 2015 and 2017 and stored at -80°C.

Tissue microarrays (TMAs) with 55 paired cases of CRC and adjacent nontumorous tissues, plus 14 individual CRC tissues, were obtained from Shanghai Tenth Hospital (Shanghai, PR China). These CRC specimens were collected from CRC patients between 2010 and 2015 and followed until April 2019. No patient received chemotherapy or radiation before surgery, and no other concurrent cancer was observed in the patients. Both the Institutional Review Boards of Shanghai Tenth Hospital and Huashan Hospital, Fudan University, approved our study in compliance with Helsinki Declaration of 1975 as revised in 1996. All patients signed the informed consent before surgical operation. The clinical stages were classified by the American Joint Committee on Cancer and Union for International Cancer Control (AJCC/UICC) classification system [14]. Overall survival (OS) is defined as the time interval between the date of surgery and death.

### 2.3. Cell Culture and Stable Cell Line Establishment

Human embryonic kidney cell line HEK293T and human colon/rectum cancer cell lines LoVo, DLD1, SW620, and HCT116 were purchased from Shanghai Institute of Biological Sciences. All cell lines were maintained in Dulbecco's modified Eagle's medium (DMEM) (Gibco, CA, USA) with 10% FBS (Gibco) at 37°C in an atmosphere of 5% CO_2_.

The *GALNT8* ORF sequence (NM_017417.2) and *GAU1* (NR_110112.1) were cloned by reverse-transcriptional PCR from human mRNA and were further integrated into the lentiviral expression vector pCDH (Addgene, #72265) to develop pCDH-GALNT8 and pCDH-GAU1 recombinant plasmid. Lentivirus with *GALNT8* or *GAU1* overexpression vector or pCDH control vehicle was packaged with packaging plasmid psPAX2 (Addgene, #12260) and envelope plasmid pMD2.G (Addgene, #12259) using Lipofectamine 2000 (Invitrogen, #11668019) in HEK293T. Stable cell lines overexpressing *GALNT8*, *GAU1*, or vehicle control were established with SW620 and HCT116 cell lines by lentivirus infection.

### 2.4. siRNA Interference

siRNAs targeting *GALNT8* (si-GALNT8), *GAU1* (siGAU1), and nonsense scramble (siNS) were purchased from Tuoran Biotech (Shanghai, China). The siRNA sequence is as follows: siGAU1-1: 5′-CCAAGAACUUCGGAAGCAUTT-3′, siGAU1-2: 5′-CCAGCUUACACGUCAGCUUTT-3′, siGALNT8-1: 5′-CUCGAUUGUUGAAGGAAAU-3′, siGALNT8-2: 5′-GCUCACAGAAUGUCUACUA-3′, and siNS: 5′-UCCTAAGGUUAAGUCGCCUC-3′. siRNA transfection of LoVo, DLD1, and their derived *GAU1*-overexpressing cells or vehicle control cells was undertaken with Lipofectamine RNAiMax (Invitrogen, #13778150). All the cancer cell line transfection was performed 48 hours before further experimental usage.

### 2.5. Cell Proliferation and Cell Cycle Assay

Cancer cell lines were seeded 1 × 10^3^ per well in the 96-well plate. The cell proliferation was assessed by Cell Counting Kit-8 (CCK8, MCE, #HY-K0301) every 24 hours for 5 days. The colony formation ability of cancer cell lines was measured by 0.1% crystal violet/methanol staining 10 days after cell seeding in six-well plates at 1 × 10^3^ per well density. Any colony that contains more than 50 cells was counted.

Cell cycle analysis was performed with Propidium Iodide (PI) staining. A total of 10^6^ cells were rinsed twice with cold PBS, then fixed with 75% ethanol overnight at -20°C, rinsed three times with PBS, and resuspended with 0.5 ml FxCycle™ PI/RNase Staining Solution (Life Technologies, #F10797). Keep the cell suspension for 15 min in the dark, and immediately subject to flow cytometry analysis on a FACSCanto system (BD Biosciences).

### 2.6. Quantitative Real-Time PCR

Trizol reagent (Invitrogen) was used for total RNA of tissues or cell extraction. Reverse transcription was performed with PrimeScript™ RT Reagent Kit (TaKaRa Biotechnology, #RR047A). Quantitative real-time PCR was conducted with TB Green Premix (TaKaRa Biotechnology, #RR820A) and gene-specific primers (Table 1) on an Applied Biosystems 7500 system (ABI); *β*-actin was used as a mRNA expression housekeeping gene (Table 1). Relative expression of *GALNT8* and *GAU1* was calculated with the 2^-*ΔΔ*Ct^ method.

### 2.7. Antibody and Regent Information

The primary antibodies are GALNT8 (Abcam, #ab121374) and *β*-actin (Cell signaling technology, #3700). The secondary antibodies are HRP-labeled goat anti-rabbit IgG (Thermo Fisher, #31460) and HRP-labeled goat anti-mouse IgG (Thermo Fisher, #31430). Oxaliplatin was purchased from MCE (#HY-17371).

### 2.8. Protein Isolation and Immunoblotting

Cancer cell samples were suspended with 0.05% trypsin and washed twice with cold PBS and, after, homogenized with RIPA lysis buffer and protease inhibitor cocktail (Beyotime Biotechnology, #P0013D) on ice for 30 minutes. Cell lysates were harvested by 4°C centrifuge and diluted with 2× SDS sample buffer. The denaturized protein samples were resolved by SDS-PAGE and transferred onto polyvinylidene fluoride (PVDF) membranes (Millipore, #ISEQ00010). Blocked with 5% skimmed milk in PBST, the PVDF membranes were incubated with specific primary antibodies overnight at 4°C. After 3 times of 10-minute TBST buffer rinsing, the membranes were again incubated with secondary antibodies for 1 hour at room temperature and rinsed 3 times with TBST buffer for 10 minutes. Signals were detected with enhanced chemiluminescence (ECL) substrate (ThermoFisher, #32106) on a Las-3000 Luminescent Image Analyzer (Fujifilm, Japan).

### 2.9. TMA Staining and Immunohistochemistry

The TMA slide was air-dried at 60°C for an hour and treated with 0.01 M citric acid buffer solution for antigen retrieval. After cooling down to room temperature, the slide was further treated by 3% H_2_O_2_ solution in methanol for 10 minutes and rinsed 3 times with cold PBS before incubation with primary anti-GALNT8 antibody (1 : 100) at 4°C overnight. The slides were rinsed three times for 5 minutes and then incubated with ready-to-use biotinylated goat anti-rabbit IgG (Abcam, #ab64256) solution for 15 minutes at room temperature, followed by PBS rinsing for five times. Streptavidin peroxidase complex (Abcam, #ab64269) was applied to the TMA and incubated for 10 minutes at room temperature and rinsed by PBS for five times. After visualization with diaminobenzidine chromogen (Abcam, #ab64238) and hematoxylin counterstaining, the TMA was imagined using a Nikon Eclipse E-800 microscope. The stained TMA was then independently reviewed by two pathologists and rated for the grade of GALNT8 staining with scores of -, +/-, +, ++, and +++.

### 2.10. Cytotoxic Assay

For SW620 and DLD1, the cells with manipulated GAU1 expression or control were seeded in the 96-well plate at a density of 1 × 10^3^ cells per well and incubated with low serum medium (1% *v*/*v* FBS) with or without oxaliplatin. Cells were replenished with fresh low serum medium with or without oxaliplatin on the third day. Cell Counting Kit-8 (CCK8, MCE, #HY-K0301) assay was used to estimate the cell viability at the end of the fifth day of treatment.

### 2.11. Subcellular Isolation

Subcellular isolation in LoVo and DLD1 cells was performed as described [15] with modification. Prepare isolation buffer (1.28 M sucrose; 40 mM Tris-HCl, pH 7.5; 20 mM MgCl_2_; and 4% Triton-X 100) and diluted isolation buffer (cold H_2_O : cold PBS : isolation buffer = 3 : 1 : 1). 10^6^ cells were suspended in 200 *μ*l diluted isolation buffer and incubated on ice for 10 min. 20 *μ*l lysate was added to 1 ml Trizol for total RNA extraction. The rest of the lysate was rotated at 4°C for 20 min and centrifuged at 2500 × g for 15 min at 4°C. Add 1 ml Trizol to the supernatant for cytoplasmic RNA extraction. Wash the pellet once, resuspend with 160 *μ*l isolation buffer, and add 1 ml Trizol for the nuclear RNA extraction. Fractionated RNAs were used for cDNA synthesis and qRT-PCR.

### 2.12. Statistical and Survival Analysis

GraphPad Prism 7 (La Jolla, CA, USA) and SPSS 20 (IBM, NY) were used for statistical analysis and graph preparation. All data are displayed as means ± SD. Two-tailed Student's *t*-test was used for assessment of differences between any two groups. Kaplan-Meier analysis was used to perform survival analysis, and the patients' survival comparison between subgroups was analyzed with log-rank test. Nonparametric Wilcoxon-Mann-Whitney test was performed for patients' clinical data analysis.

## 3. Results

### 3.1. Identification of *GAU1* as the Colorectal Cancer-Related lncRNA

**Figure 1:**
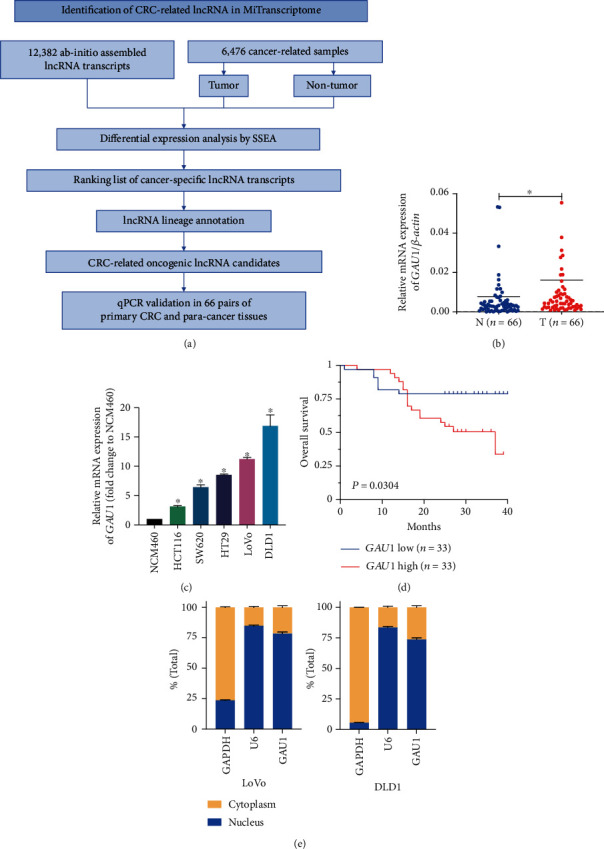
Identification of GAU1 as the colorectal cancer-related lncRNA. (a) The working pipeline of identifying CRC-related lncRNA in the MiTranscriptome database. (b) Relative expression level of *GAU1* detected by qRT-PCR in 66 paired colorectal cancer (CRC) tissues and adjacent normal tissues (*P* = 2.53 × 10^−2^). N: adjacent normal tissues; T: tumor tissues. (c) qRT-PCR analysis of *GAU1* expression in the five CRC cell lines and the normal NCM460 cells was tested. ^∗^*P* < 0.05. (d) Kaplan-Meier analysis of the correlation between *GAU1* mRNA expression and overall survival in 66 CRC patients (*P* = 3.04 × 10^−2^). (e) Total RNA from LoVo and DLD1 cells was separated into cytoplasmic and nuclear fractions and analyzed by qRT-PCR. GAPDH serves as a positive control for cytoplasmic gene expression, and U6 as a positive control for nucleolus separation.

To identify the colorectal cancer-related lncRNA in the MiTranscriptome database, we first performed the differential expression analysis for all the 5,724 cancer libraries vs. 752 noncancer libraries by SSEA and annotated all the transcripts with tissue-type information (Figure 1(a)). After the empirical ranking test, two transcripts of *GAU1* (ENSG00000255474) were listed on the top CRC-related lncRNA (ranking percentile = 99.75% of 12,382) besides our previously reported CRC-specific lncRNA *PHiL* [9] (ranking percentile = 99.62%).

Then, we further experimentally quantified the *GAU1* mRNA overexpression in human colorectal cancer cell lines SW620, HCT116, DLD1, and LoVo versus human intestinal epithelial cell line NCM460 (*P* < 0.05, Figure 1(b)). Furthermore, qRT-PCR of *GAU1* mRNA in 66 pairs of CRC tissues and adjacent normal tissues also confirmed *GAU1* as the cancer-specific lncRNA in CRC (*P* = 2.53 × 10^−2^, Figure 1(c)). More importantly, the Kaplan-Meier analysis revealed that patients with higher *GAU1* expression had worse prognosis (*P* = 3.04 × 10^−2^, Figure 1(d)), indicating *GAU1* may play an oncogenic role in CRC. And the subcellular localization showed that GAU1 was mainly distributed in the nucleus (Figure 1(e)).

### 3.2. *GAU1* Overexpression Facilitates CRC Cell Proliferation by Promoting Cell Cycle

**Figure 2:**
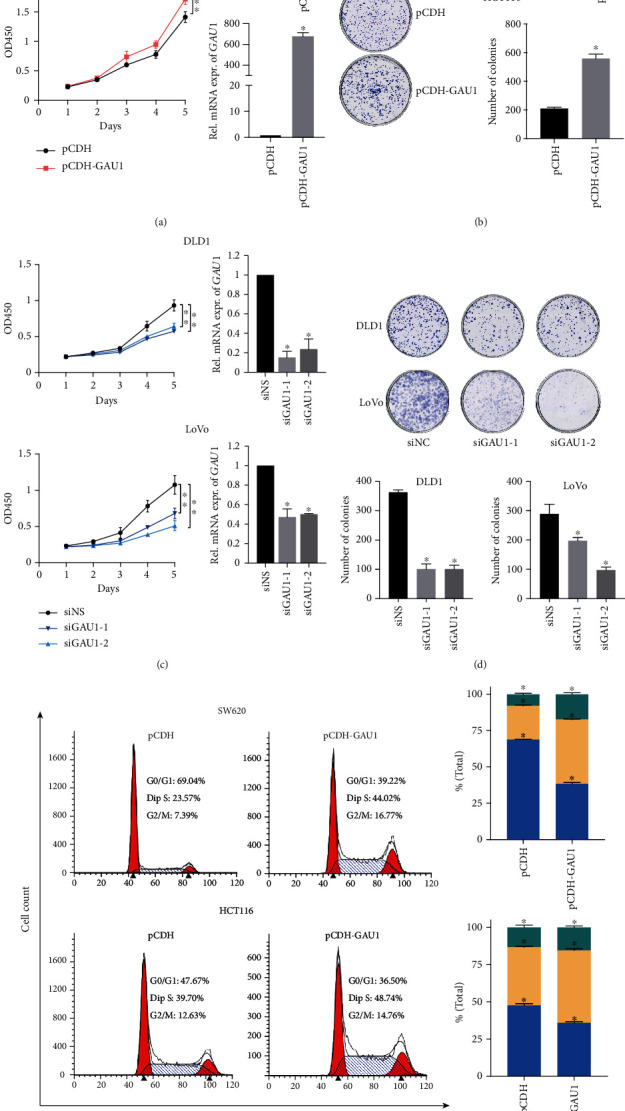
*GAU1* overexpression facilitates CRC cell proliferation. (a) CCK-8 assay and (b) colony formation assay were conducted in SW620 and HCT116 cells transfected with *GAU1* overexpression or control plasmids. ^∗^*P* < 0.05; ^∗∗^*P* < 0.01. (c) CCK-8 assay and (d) colony formation assay showed the proliferation of control siRNA (siNS) or siRNAs (siGAU1-1 and siGAU1-2) against GAU1-transfected DLD1 and LoVo cells. ^∗^*P* < 0.05; ^∗∗^*P* < 0.01. (e) Cell cycle analysis of GAU1-overexpressed HCT116, SW620, and their controls.

To further determine if the *GAU1* overexpression can alter the biological phenotype of CRC, we first established the *GAU1*-overexpressing stable cell lines by lentiviral infection of pCDH-GAU1 in SW620 and HCT116 cell lines with intermediate *GAU1* expression. The CCK-8 and clonogenic assays both revealed that *GAU1* overexpression lead to a significantly increased cell proliferation in the CRC cell lines compared to the vehicle controls (Figures 2(a) and 2(b)). Consistently, *GAU1* knockdown in the *GAU1* high-expressing LoVo and DLD1 cell lines by short interfering RNA (siRNA) significantly reduced the cell proliferation and clonogenic ability of the CRC cells (Figures 2(c) and 2(d)). These data suggested that *GAU1* overexpression promotes CRC cell proliferation in vitro. Moreover, the cell cycle profile alteration after *GAU1* overexpression (increased S-phase commitment) (Figure 2(e)) also implied *GAU1* as a critical player in promoting S-phase entry.

### 3.3. *GALNT8* as the Oncogenic Operator of *GAU1* in CRC

**Figure 3:**
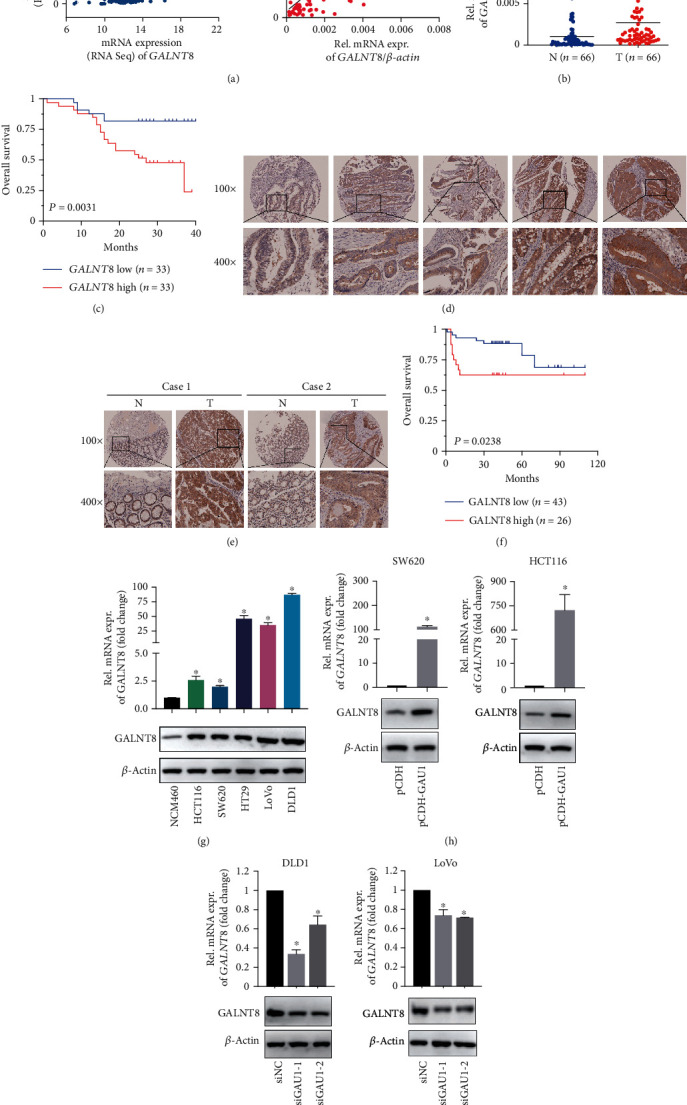
GALNT8 as the oncogenic operator of *GAU1* in CRC. (a) Correlation analysis of *GALNT8* and *GAU1* in 184 TCGA CRC samples (Spearman rho = 0.67, *P* = 2.44 × 10^−23^), and 66 frozen CRC tissues (qRT-PCR, *R*^2^ = 0.41, *P* < 10^−4^). (b) qRT-PCR analysis of *GALNT8* expression level in 66 paired CRC tissues and adjacent normal tissues (*P* < 10^−4^). N: adjacent normal tissues; T: tumor tissues. (c) Kaplan-Meier analysis of *GALNT8* and overall survival in 66 CRC patients. (Kaplan-Meier *P* = 0.31 × 10^−2^). (d) Representative photomicrographs of GALNT8 in CRC specimen TMA (magnification: ×100, ×400). (e) Two paired tumor-adjacent control representative cases of GALNT8 expression in the TMA (magnification: ×100, ×400). (f) Kaplan-Meier analysis of *GALNT8* expression and overall survival in 69 TMA samples. (Kaplan-Meier *P* = 2.38 × 10^−2^). (g) qRT-PCR and western blot analysis of *GAU1* expression in the five CRC cell lines and the normal NCM460 cells was tested. ^∗^*P* < 0.05. (h) qRT-PCR and western blot analysis of GALNT8 expression level in *GAU1*-overexpressed SW620 and HCT116 cells, as well as (i) *GAU1*-knockdown DLD1 and LoVo cells. ^∗^*P* < 0.05.

To identify the biological “operator” of *GAU1* overexpression in CRC development, we performed coexpression analysis for *GAU1* in 184 TCGA CRC samples. Correlation analysis revealed *GALNT8*, located in the vicinal gene loci of *GAU1* on chromosome 12, as the most significantly coexpressed gene of *GAU1* among all the 19,815 protein-coding genes (Spearman rho = 0.67, *P* = 2.44 × 10^−23^, Figure 3(a)). The strong expression correlation between *GAU1* and *GALNT8* was further validated in our 66 pairs of clinical samples (*P* < 10^−4^, Figure 3(a)), with a significant upregulation of *GALNT8* expression in the tumor tissues (T) compared with the adjacent nontumorous tissues (N) (*P* < 10^−4^, Figure 3(b)). Clinically, the Kaplan-Meier analysis also revealed that patients with higher GALNT8 expression had worse overall survival (*P* = 0.31 × 10^−2^, Figure 3(c)).

The overexpression of GALNT8 in the CRC patients was further validated by the IHC staining of a TMA containing 55 paired cases of CRC and adjacent nontumorous tissues, plus 14 individual CRC tumors. According to the density of IHC staining (Figure 2(d)), GALNT8 protein expression in tumor tissues was classified as high expression (score ++, score +++) in 26 cases (26/69, 37.68%) and low expression (score +, score +/-, and score -) in 43 cases (43/69, 62.32%) (Figure 3(d)). Tumor tissues harbored a significantly increase GALNT8 expression compared to the adjacent nontumorous tissues (Fisher exact *P* = 3.30 × 10^−8^, Figure 3(e)). Further survival assay by Kaplan-Meier analysis also revealed that CRC patients with overexpressed GALNT8 suffered from poor overall survival (*P* = 2.38 × 10^−2^, Figure 3(f)).

Moreover, in contrast to human intestinal epithelial cell line, a higher expression of GALNT8 in CRC cells was observed in both mRNA and protein levels (Figure 3(g)). To further confirm the regulatory effect of *GAU1* on *GALNT8* expression, the effect of *GAU1* knockdown/overexpression on the expression levels of GALNT8 in CRC cells was determined. The mRNA and protein expression levels of GALNT8 were increased in the *GAU1* overexpression cell lines and decreased in the siGAU1 cell lines compared with the control group (Figures 3(h) and 3(i)). Altogether, the computational and experimental evidence suggested GALNT8 as a regulatory downstream molecule of *GAU1* in CRC.

### 3.4. The Oncogenic Ability of *GAU1* Is GALNT8 Dependent

**Figure 4:**
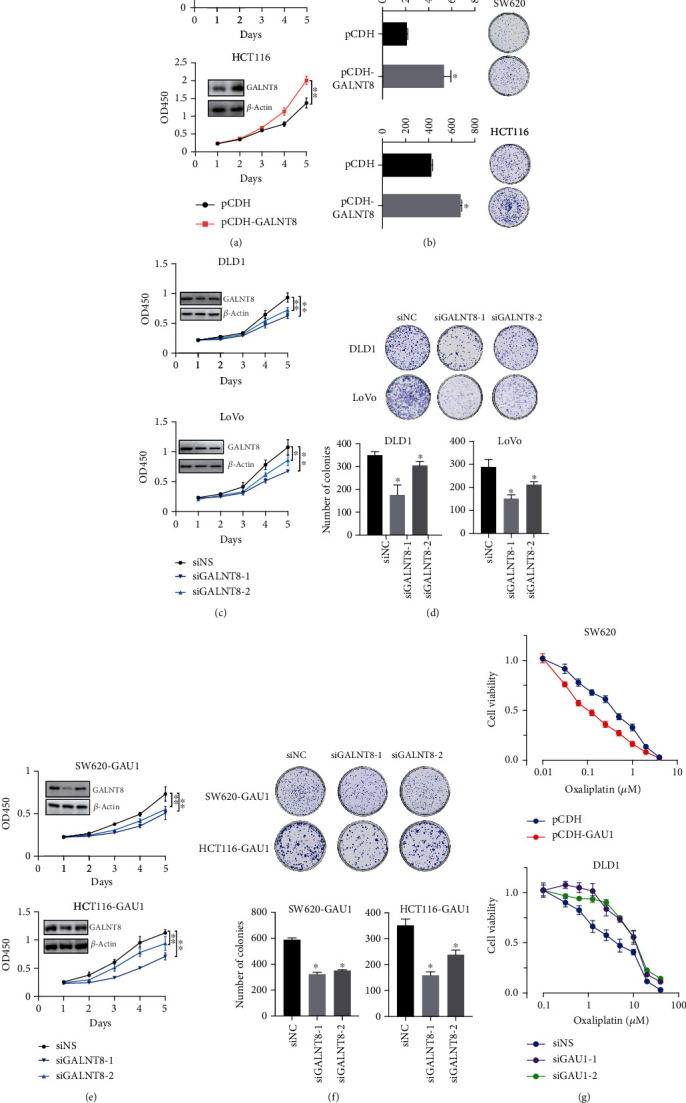
The oncogenic ability of *GAU1* is GALNT8 dependent, and overexpression of GAU1/GALNT8 axis sensitizes CRC cell lines to chemotherapy. (a) CCK-8 assay and (b) colony formation assay were used to measure proliferation in SW620 and HCT116 cells transfected with pCDH-GALNT8 or pCDH. ^∗^*P* < 0.05; ^∗∗^*P* < 0.01. (c) CCK-8 assay and (d) colony formation assay were conducted in control siRNA (siNS) or siRNAs (siGALNT8-1 and siGALNT8-2) against GALNT8-transfected DLD1 and LoVo cells. ^∗^*P* < 0.05; ^∗∗^*P* < 0.01. (e, f) Proliferation analysis by (e) CCK-8 assay and (f) colony formation assay in *GAU1*-overexpressed SW620 and HCT116 cells transfected with control siRNA (siNS) or siRNAs (siGALNT8-1 and siGALNT8-2) against GALNT8. ^∗^*P* < 0.05; ^∗∗^*P* < 0.01. (g) Viability of pCDH-GAU1- or pCDH-transfected SW620 and siGAU1-1/2- or siNS-transfected DLD1 cells incubated with multiple concentrations of oxaliplatin (0.01-40 *μ*M) was monitored through CCK-8 assay.

Since the relationship between GALNT8 and cancer is limited, we experimentally manipulated the expression of *GALNT8* by lentiviral stable overexpression and siRNA interference. CCK-8 and colony forming assays demonstrated that the overexpression of *GALNT8* enhanced the proliferation and colony formation capacity of SW620 and HCT116 (Figures 4(a) and 4(b)), whereas the contrary results were observed in the GALNT8-suppressed DLD1 and LoVo cell lines (Figures 4(c) and 4(d)). With all these results, it is suggested that *GALNT8* contributes to CRC cell proliferation.

To further explore the oncogenic partnership of the *GAU1*/*GALNT8* cluster in CRC, siGALNT8 or negative control was transfected into *GAU1*-overexpressing cell lines to examine whether *GALNT8* silence could rescue *GAU1* overexpression-mediated enhanced proliferation of CRC. The CCK-8 and colony formation assay results demonstrated that the upregulated cell proliferation and colony formation in the *GAU1*-overexpressed SW620 and HCT116 cell lines were partially attenuated by siGALNT8 in Figures 4(e) and 4(f)), suggesting that *GALNT8* is a critical downstream operator of *GAU1* during the CRC proliferation.

### 3.5. Overexpression of *GAU1*/GALNT8 Axis Sensitizes CRC Cell Lines to Chemotherapy

Given the experimental evidence that *GAU1*/GALNT8 axis overexpression significantly promotes the cancer cell proliferation and *GAU1* boosts cell cycle by increasing S-phase entry, we further questioned if *GAU1*/GALNT8 axis can reshape the drug response of cancer cells to chemotherapy agents targeting DNA replication. The *in vitro* oxaliplatin drug response data revealed that cancer cells overexpressing *GAU1* or *GALNT8* are more vulnerable to chemotherapy agents causing replication fork collapse, indicating *GAU1*/GALNT8 axis as a potential actionable target for the personalized medicine of CRC. This finding was further confirmed by the drug resistance phenotype in *GAU1*/*GALNT8* knockdown cell lines (Figure 4(g)).

## 4. Discussion

CRC is one of the most common and lethal types of cancer [16]. In the past decades, genetic alteration including *APC* and *K*-*RAS* somatic mutation has been identified to cause 70% of the CRC cases [17] and widely adapted into the diagnosis and drug response prediction during CRC management [18].

Recent studies attributed the transcriptional alteration of the lncRNAs as a hallmark of tumor development [19, 20]. The enormous efforts on the landscaping of lncRNA expression in cancer [21, 22] led to a number of fabulous investigations that improved the understanding of multiple major cancer types [7].

In this study, we identified *GAU1* as one of the major oncogenic lncRNAs for CRC by mining the ab initial strategy-based lncRNA database MiTranscriptome [10, 11]. According to our analysis, *GAU1* ranked one of the most differentially expressed lncRNAs between CRCs and normal tissues/cell lines (99.75% percentile of SSEA). Moreover, the overexpression of *GAU1* leads to a significant reduction in CRC patient survival (*P* = 3.04 × 10^−2^). After experimentally validating the procancerous ability of *GAU1* by the cell proliferation assay after *GAU1* expression manipulation in CRC cell lines, we further located *GALNT8* as the mostly coexpressed protein-coding gene for *GAU1*.

*GALNT8* encodes a 637-amino-acid type-II membrane protein (GalNAc-T8) [23]. The protein is a member of the UDP-GalNAc polypeptide N-acetylgalactosaminyl transferase (ppGaNTase) family, which initiates mucin-like O-linked protein glycosylation in the Golgi apparatus [24]. Previous research revealed that GALNT8 is expressed in the heart, placenta, skeletal muscle, liver, and kidney and plays a key role during embryonic development [23]. However, the oncogenic effect of GALNT8 is less characterized. Chai et al. reported *GALNT8* as the oncogene in retinoblastoma that potentially drives the cancer development and progression [25] by directly binding to the *GALNT8* promoter and boost the transcription of *GALNT8* through TCEA1 (Transcription Elongation Factor A1) recruitment, which mechanistically endorsed our experimental data in CRC.

Like *GAU1*, *GALNT8* is also associated with poor CRC prognosis (*P* = 0.31 × 10^−2^). Together with the experimental evidence (1) overexpression or silencing *GALNT8* mimicked the cancer cell line phenotypic alteration after *GAU1* overexpression or knockout. (2) *GALNT8* knockdown attenuated the *GAU1* overexpression-induced cell proliferation, and not vice versa; we confirmed GALNT8 as the downstream operator of *GAU1* in CRC.

Aside from the surgical operation, systemic chemotherapy with folinic acid, fluorouracil, and oxaliplatin (FOLFOX) is also a main treatment solution for CRC. Our result showed an oxaliplatin hypersensitivity in cancer cell lines overexpressing *GAU1*/GALNT8. This double-edge sword effect of *GAU1*/GALNT8 overexpression suggested the *GAU1*/GALNT8 axis as a potential marker in the precision medicine of CRC, although more experimental evidence should be investigated in the future.

One limitation of this study is we did not provide the molecular interaction between *GAU1* and *GALNT8*. Although we have confirmed GALNT8 as the essential operator for the oncogenic ability of *GAU1*, further investigation on the regulatory mechanism between these bidirectionally transcribed lncRNA/protein-coding gene pairs needs to be clarified by protein-RNA interaction or DNA-RNA binding assay. According to the previous report that *GAU1* and *GALNT8* share a cisregulation relationship in retinoblastoma [25] and the mutual promoter region of the two genes, investigation on the mechanism behind the abnormal promoter activation in CRC should be conducted in our future studies.

To our best knowledge, this is the first study systematically reporting the oncogenic cascade of *GAU1*/*GALNT8* axis in CRC. By integrating the differential expression data from 7,256 curated RNA-Seq libraries in MiTranscriptome and experimental validation, we demonstrated that *GAU1*, together with its downstream protein GALNT8, is associated with cancer cell proliferation, poor patient survival, and chemotherapy response.

## Figures and Tables

**Table 1:** Primer sequences for gene amplification.

Gene	Strand	Sequences (5′-3′)
GALNT8	Forward	ACGCCCTCTCGATTGTTGAA
	Reverse	CTCTGCCCACCCAACATTGA

GAU1	Forward	GCCCTTCCCAAAGCACAAAT
	Reverse	AGCACGTTAAGAGGCTTGGA

*β*-Actin	Forward	TTGTTACAGGAAGTCCCTTGCC
	Reverse	ATGCTATCACCTCCCCTGTGTG

## Data Availability

The data used to support the findings of this study are included within the article.
